# Closing the know-do gap for child health: UNICEF’s experiences from embedding implementation research in child health and nutrition programming

**DOI:** 10.1186/s43058-021-00207-9

**Published:** 2021-09-29

**Authors:** Debra Jackson, A. S. M. Shahabuddin, Alyssa B. Sharkey, Karin Källander, Maria Muñiz, Remy Mwamba, Elevanie Nyankesha, Robert W. Scherpbier, Andreas Hasman, Yarlini Balarajan, Kerry Albright, Priscilla Idele, Stefan Swartling Peterson

**Affiliations:** 1 Implementation Research and Delivery Science Unit, Health Section, Programme Division, UNICEF, New York, New York, USA; 2 Takeda Chair in Global Child Health, London School of Hygiene and Tropical Medicine, Keppel Street, London, UK; 3 School of Public Health, University of the Western Cape, Cape Town, South Africa; 4 Health Section, East and Southern Africa Regional Office, UNICEF, Nairobi, Nairobi, Kenya; 5 Nutrition Section, Programme Division, UNICEF, New York, New York, USA; 6 Office of Research Innocenti, UNICEF, Florence, Florence, Italy; 7 Office of the Associate Director for Health, Programme Division, UNICEF, New York, New York, USA; 8 Uppsala University, Women’s and Children’s Health (IMCH) and Karolinska Institutet, Uppsala, Sweden; 9 Makerere University School of Public Health, Kampala, Uganda

**Keywords:** Embedded implementation research, UNICEF, Child health and well-being

## Abstract

UNICEF operates in 190 countries and territories, where it advocates for the protection of children’s rights and helps meet children’s basic needs to reach their full potential. Embedded implementation research (IR) is an approach to health systems strengthening in which (a) generation and use of research is led by decision-makers and implementers; (b) local context, priorities, and system complexity are taken into account; and (c) research is an integrated and systematic part of decision-making and implementation. By addressing research questions of direct relevance to programs, embedded IR increases the likelihood of evidence-informed policies and programs, with the ultimate goal of improving child health and nutrition.

This paper presents UNICEF’s embedded IR approach, describes its application to challenges and lessons learned, and considers implications for future work.

From 2015, UNICEF has collaborated with global development partners (e.g. WHO, USAID), governments and research institutions to conduct embedded IR studies in over 25 high burden countries. These studies focused on a variety of programs, including immunization, prevention of mother-to-child transmission of HIV, birth registration, nutrition, and newborn and child health services in emergency settings. The studies also used a variety of methods, including quantitative, qualitative and mixed-methods.

UNICEF has found that this systematically embedding research in programs to identify implementation barriers can address concerns of implementers in country programs and support action to improve implementation. In addition, it can be used to test innovations, in particular applicability of approaches for introduction and scaling of programs across different contexts (e.g., geographic, political, physical environment, social, economic, etc.). UNICEF aims to generate evidence as to what implementation strategies will lead to more effective programs and better outcomes for children, accounting for local context and complexity, and as prioritized by local service providers. The adaptation of implementation research theory and practice within a large, multi-sectoral program has shown positive results in UNICEF-supported programs for children and taking them to scale.

Contributions to the literature

1. Embedded implementation research (IR) is an approach to support health systems strengthening in which research is made integral to decision-making for program improvement. There are a variety of approaches and frameworks for embedded and implementation research described in the literature, but none specifically highlight use by a large multi-lateral organization as an approach globally to address program challenges and bottlenecks.
2. UNICEF’s mandate is to protect, promote, and fulfil children’s rights. The adaptation of implementation research theory and practice within UNICEF, a large, multi-sectoral organization, has shown positive results for improving programs for children. Since 2015, UNICEF has worked in collaboration with global development partners, governments and research partners to conduct embedded IR studies in over 25 high burden countries. This paper highlights the approach taken by a large multi-lateral organization to embed implementation research to improve policy and programming for children.

## Introduction: need for actionable knowledge to improve programs for children

Significant progress in maternal and child health has been achieved over recent decades. Global under-five mortality dropped by more than half since 1990 [1]. Global maternal mortality fell 38% since 2000 [2]. Despite these achievements, unacceptable inequities in intervention coverage and child mortality remain, both among and within countries. Attention is needed to improve the quality of health and nutrition services and address systems challenges. Also, the contexts in which children live are changing [3, 4]. In 2030, children will live in a world that is more urban, mobile, interconnected, and with an aging population. Income growth will shift some children into wealthier, but not necessarily healthier environments. Fragility is also expected to persist in countries struggling with extreme poverty, conflict, and weak governance. Emergencies, including public health emergencies and those stemming from environmental causes and climate change, are expected to increase in frequency [5].

Leroy et al. [6] noted that research on development of new interventions in child health and nutrition could potentially reduce under-five child mortality by 22%, whereas if existing proven interventions were fully implemented, these programs could reduce under-five mortality by 63%. They note the paradox that the majority of research funding focuses on new interventions (97%), rather than addressing implementation challenges (3%). This paradox demonstrates the urgent need to focus on implementation research (IR) to identify barriers and effective strategies to implementation of existing proven interventions. Evidence of the effect of long-term consistent investments in embedded IR on improved service coverage and efficient use of routine health system resources has recently emerged from Ghana [7, 8] and Latin America and the Caribbean [9].

This paper presents UNICEF’s embedded IR approach, its application to maternal, child and nutrition programs, and present experiences to date; describes challenges and lessons learned; and considers implications for future work. The authors are all UNICEF staff who have developed and implemented the approach across the organization.

## Embedded implementation research

Implementation research is part of the broader field of implementation science. Rapport et al. define Implementation Science as “the scientific study of methods translating research findings into practical, useful outcomes,” but also note that the science is currently “contested and complex” [10]. Eccles and Mittman in the launch of the journal *Implementation Science* defined implementation research as “the scientific study of methods to promote the systematic uptake of research findings and other evidence-based practices into routine practice, and, hence, to improve the quality and effectiveness of health services and care” [11], while Peters et al. [12, 13] note that “The basic intent of implementation research is to understand not only what is and isn’t working, but how and why implementation is going right or wrong, and testing approaches to improve it.” This form of research addresses implementation bottlenecks, identifies optimal approaches for a particular setting, and promotes the uptake of research findings. Further, Ghaffar and colleagues [14] argued that IR should be ‘embedded’ in programming in partnership with policymakers and implementers, integrated in different settings and take into account context-specific factors to ensure relevance in policy priority-setting and decision-making. This view is further supported by Langlois and colleagues [9]. Churuca et al. note that “Embedded implementation research involves a knowledgeable researcher working with, or within, the team responsible for change, adoption, or take-up” and go on to describe four approaches of “embedded” research: dichotomized research-practice, collaborative linking-up, partially embedded, and deep immersion, describing the researcher-implementer relationship [15].

Varying definitions of operational, implementation and health systems research often cause confusion for both researchers and program managers. For example, the distinction between operations research and IR has been debated [16] as the two types of research are often similar in intent and scope. Definitions are often progressive without clear delineations, so many times operational, implementation, and health system research overlap [17]. Similar issues exist across other terms, such as formative research, process evaluation, and translational research.

## UNICEF’s approach

Working in 190 countries and territories, UNICEF advocates for the protection of children’s rights, meets children’s basic needs, and expands their opportunities to reach their full potential. UNICEF’s comparative advantage is to work across sectors and across the life-cycle to protect these rights, focusing particularly on protecting the most disadvantaged and vulnerable children. UNICEF’s strategic plan 2018-20219 describes research as a “how” strategy to achieve targets [18]. UNICEF notes that in some situations while substantial evidence exists on what needs to be done, there are evidence gaps when it comes to identifying viable approaches for sustainable, full implementation and scale-up [19] of responses to improve programs for children.

As a large multi-lateral organization with a primary mandate of improving programs for children, UNICEF approached implementation research based on practical approaches and frameworks from the literature, with a focus on ‘how’ as defined in the strategic plan. UNICEF uses definitions outlined by Remme et al. [17], where operational research is a subset of IR, which is a subset of broader health systems research. In addition, UNICEF has primarily adapted taxonomy as described by Peters et al. and others [12–14] and has further defined embedded IR as “the integration of research within existing program implementation and policymaking to improve outcomes and overcome implementation bottlenecks,” and primarily uses either the” collaborative linking-up” or “partially-embedded” approaches for establishing the relationship of researcher and implementer as defined by Churucca et al. [15] UNICEF is adapting these innovative approaches for health systems strengthening in which research is made integral to decision-making. It includes (a) positioning research within existing programs and systems, building a new evidence ecosystem, and drawing siloed sectors together; (b) meaningful engagement and leadership roles for decision-makers and implementers within the research team; and (c) when possible, aligning research activities with program implementation cycles. Embedding increases the likelihood of evidence-informed policies and programs. Embedding IR into the policymaking and systems strengthening process amplifies ownership of evidence, recognizes decisions are not made on evidence alone, and also takes societal context and values into account.

Peters et al. note that research on innovations encompasses basic science through translation to sustainable implementation of new or existing evidence-based programs [12]. IR in UNICEF focuses on the latter stages of this continuum, i.e., issues ranging from how a program works in real-world settings to systems integration and scale-up, including decision-making, policy development, and creating an enabling environment for implementation.

**Fig. 1:**
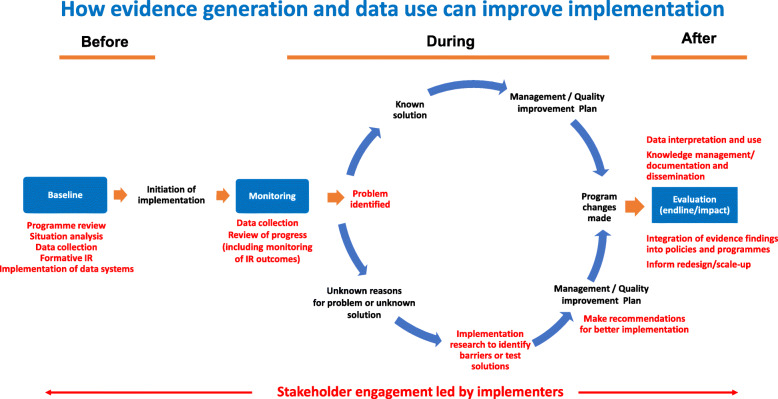
Where implementation research fits in program knowledge and learning cycle

IR to support UNICEF programs is generally located within monitoring, evaluation, and knowledge translation or learning, as a basis for program innovation, systems strengthening and implementation. IR does not take the place of routine data collection, monitoring and evaluation, but is a complement to it. Figure 1 provides a conceptual model for how IR fits into the overall evidence and learning cycle for program management. Implementation research can also be associated quality improvement programming as part of learning and evaluation [20–22]. UNICEF uses IR at various stages of program implementation, including formative and initial implementation stages and throughout the program cycle. We see IR as particularly useful for understanding challenges or bottlenecks which might be found while monitoring implementation of the program. This is a practical applied adaptation of IR suited for the needs of UNICEF. Recognizing that implementation science and implementation research has a variety of approaches and methods [12–16], UNICEF sought to apply IR within our program support cycles in collaboration with governments and implementing partners. While the broader field of implementation science is critical, we needed to adapt the approach to fit the targeted and very focused needs of our country programs.

## Embedded implementation research at UNICEF: experiences 2015–2019

UNICEF, in partnership with the Alliance for Health Policy and Systems Research (The Alliance) and the Special Program for Research and Training in Tropical Diseases (TDR), has adapted an embedded IR approach for systems strengthening for country-based programs for children. As part of the UNICEF Health Systems Strengthening Approach [23], the research seeks to catalyze a shift in the way evidence is generated and used within countries to inform policy and decision-making. By bringing together (a) in-country decision-makers at national, sub-national, and local levels; (b) country-based researchers; and (c) global development partners, it puts local decision-makers and implementers in the driving seat in the research process, while identifying clear roles for different stakeholders.

The UNICEF approach aims to enhance ownership of the research among local implementers, similar to the co-production and collaborative approach to health systems research recently highlighted by Redman et al. on behalf of the “Co-production of Knowledge Collection Steering Committee” [24]. Although it may influence local or higher-level policy, it is primarily designed to prioritize research on questions of local relevance, build capacity to conduct local IR to generate feasible recommendations in “real-time” and underwrite policy and system strengthening. IR can also be considered during program initiation to answer questions on the acceptability, appropriateness and feasibility of alternative delivery strategies, as well as blending with evaluation, using “effectiveness-implementation hybrid designs” [12, 25], to address program effectiveness.

UNICEF works with both global and local partners to identify priorities on implementation barriers needing resolution through systematic and inclusive IR processes. Programs, through IR, can learn why implementation barriers and contextual variances mean that interventions work well in one context, but not in another. In addition, it can be used to test new approaches or innovations from pilot through scale-up across different contexts (e.g., geographic, political, physical environment, social, economic). Implementation research can also document failures, where an intervention success could not be replicated given local context, which is equally valuable to prevent wastage of funds before investing in scale-up.

**Fig. 2:**
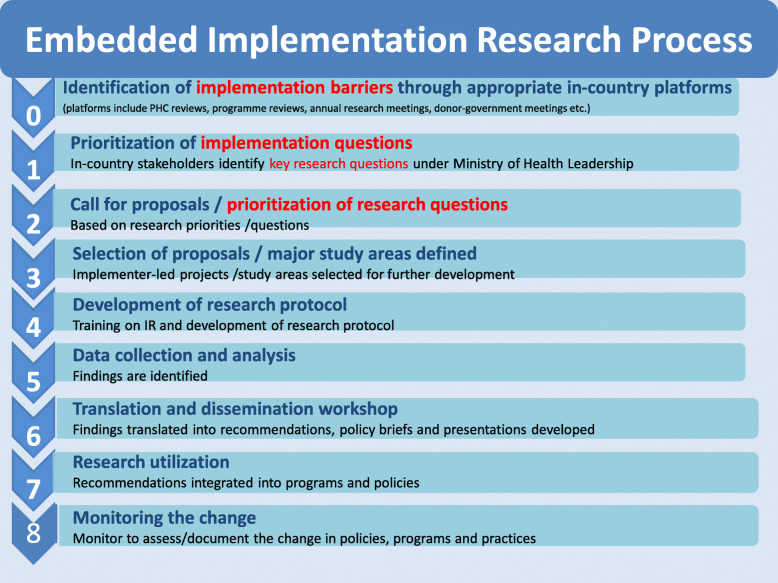
UNICEF embedded implementation research process

UNICEF’s embedded IR approach generally starts with sensitization of national stakeholders including Ministry of Health and policymakers to what IR is and what the potential benefits are (Table 1). Implementation barriers, often previously identified through national, sub-national, or local program reviews, or monitoring and evaluation, are reviewed, summarized, and prioritized. In collaboration with national stakeholders and policymakers, implementation barriers are then transformed into priority research questions and potential related IR studies are identified. A research team comprising a partnership between national policy-makers, local decision-makers, and implementers (e.g. program managers, district managers, front-line health workers), and in-country researchers is convened. Through this process, UNICEF staff, in partnership with implementation researchers or research institutions (global or local), provide technical support and training to develop protocols, ensure ethical research standards are maintained, conduct studies, and support communication of results, recommendations, and use of the findings for policy and program changes. Figure 2 provides an example of the UNICEF embedded IR process.

**Table 1:** Key characteristics of UNICEF’s embedded implementation research approach

➢ Context specific—community, district, national ➢ Culturally sensitive, taking into account religious and cultural norms ➢ Relevant policy- and agenda-setting purpose—addresses the foundations of policy and challenges to implementation ➢ Methods fit for purpose—range of designs ➢ Demand driven—needs identified by policymakers, implementers or consumers (e.g. adolescents) ➢ Multi-stakeholder and multi-disciplinary - not just health ➢ Real world—usually under implementation rather than controlled trial or study conditions ➢ Real time—aligned with policy and program cycles and in time for real time improvements and adaptation ➢ Focuses on processes and outcomes—documents what is feasible and how ➢ Tacit knowledge is used and acknowledged, and lesson-learning is embedded within the intervention—**cannot do implementation research without the implementers, preferably IR is embedded in programs and led by implementers.**	

Adapted from Peters et al. [12]

**Fig. 3:**
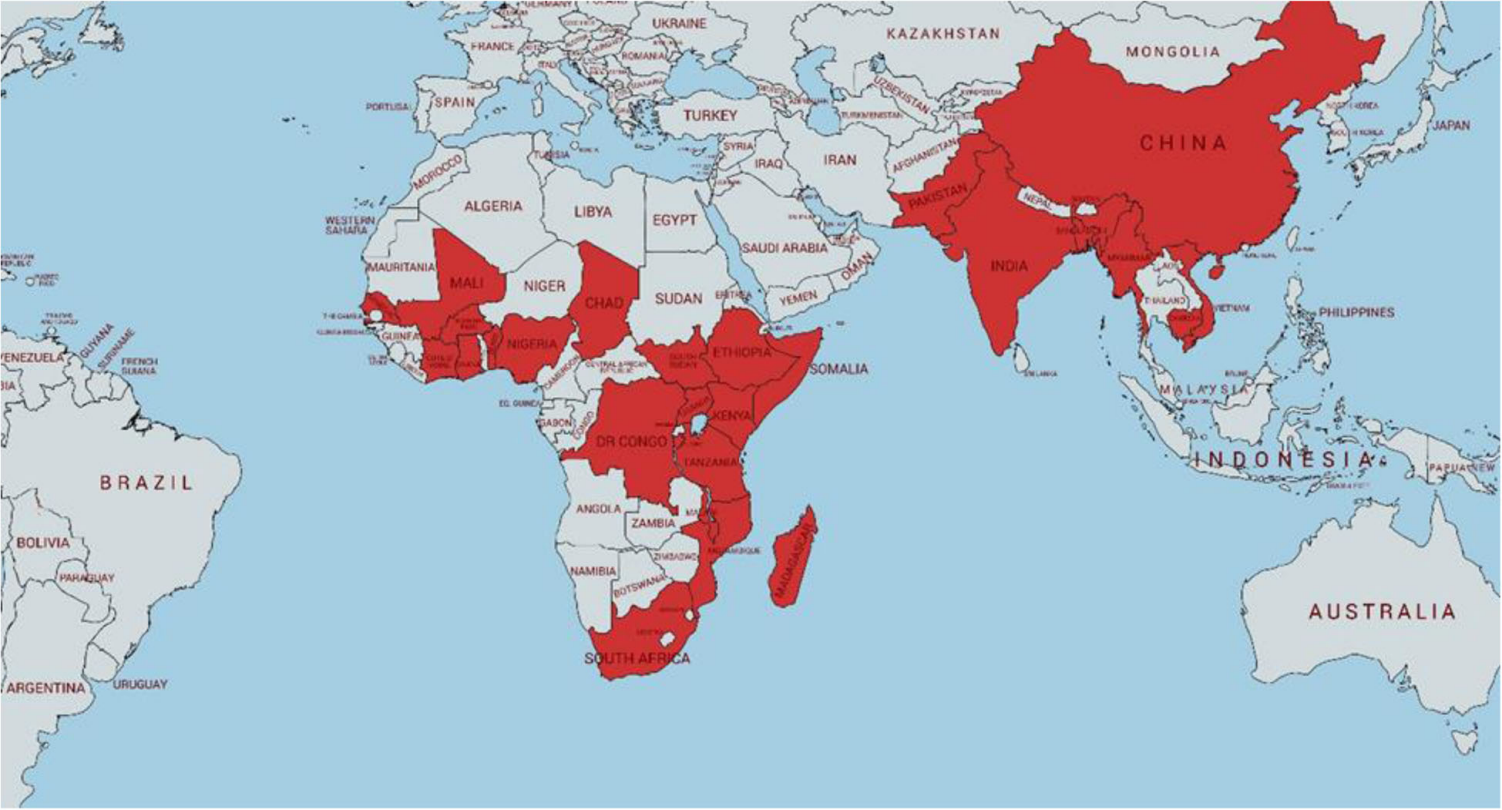
Countries participating in UNICEF-supported embedded implementation research since 2015

Figure 3 shows countries where UNICEF has worked with partners to embed IR within existing programming activities. In collaboration with in-country researchers, policy-makers and program implementers, UNICEF has been supporting IR projects globally since 2015. The research has varied from formative early stages of programming, through initial implementation, to full implementation. Methodological approaches varied by study and included quantitative, qualitative, and mixed-methods. Study questions have addressed a wide variety of multi-sectoral topics (e.g., immunization, child health days, birth registration, newborn and child health in humanitarian settings, prevention of mother-to-child transmission of HIV) and health system challenges (e.g., information systems, human resources, supply chain, demand for services, community engagement, integration).

### Measuring embedded implementation research success

UNICEF’s selected measure of success for embedded IR is that the research findings are used for policy and/or program changes. This near-real-time use of findings is key. Dissemination and publication of the results alone does not count as “use,” and policy or program changes should be documented, no matter how small or local. Selected examples can be found in Table 2 Case studies. Informal feedback suggests that from two-thirds to three-quarters of the IR studies successfully resulted in a documented policy or program change. Reasons for lack of use of findings suggested were limited time and resources and lack of formal follow-up after the research.

**Table 2:** Case study examples of UNICEF implementation research

**Case study 1: India** Negative social media messages on vaccines: How can the resultant trust deficit between caregivers and health workers be overcome? A qualitative inquiry in Malappuram district of Kerala State in India. This study partnered the Kerala Ministry of Health Vaccination Program with a research consultant as part of a joint WHO-UNICEF GAVI supported multi-country program Decision-Maker Led Implementation Research (DELIR) Initiative [17]. The focus of this research was not on developing and testing new interventions, but rather the generation of strategies and knowledge that will enable more effective implementation of existing immunization programs. The projects supported under DELIR address various aspects of system failures, implementation barriers and implementation strategies relating to immunization coverage and equity. Each immunization program developed their own priority question and was supported from protocol development through dissemination and program action. In Kerala State, India, the immunization team was facing an increasing problem of anti-vaccination messaging in the community and aimed to understand this messaging and how to combat it. *Research objectives:* 1. Understand the role of anti-vaccine social media messages in influencing the relationship and trust between caregivers and health workers. 2. Understand how the deficit in caregivers’ trust in health workers influences their decisions on childhood vaccination. 3. Evaluate the current communication methods (e.g., information, education and communication (IEC) materials, websites etc.) vis-à-vis the ability to address anti-vaccine messages. 4. Suggest modifications in the current communication and social media activities to improve the trust between caregivers and health workers and thereby improve vaccination coverage. 5. Develop a set of tools for health workers (leaflets, FAQs, social media messages) and educational materials which can help to counter anti-vaccine messages. *Methods:* Qualitative key informant interviews and focus group discussions with immunization program staff and children’s caregivers, plus desk review of current IEC materials. *Results:* Examined how to counter anti-vaccine propaganda leading to a revised communication strategy, including a mobile social media app to target the primary platform for anti-vaccine messaging [26]. **Case study 2: Malawi** Research to explore adolescent needs and barriers to service uptake and retention for pregnant and postpartum HIV-positive adolescents in Malawi’s PMTCT program This project was part of a multi-country UNICEF program, funded by Sweden, to address retention of postpartum mothers and babies in prevention of mother-to-child transmission of HIV (PMTCT) program in four countries. After 2 years of implementation each country team identified in collaboration with the ministry of health and implementing partner a priority challenge facing the implementation of the retention program. In Malawi, the ministry had decided to prioritize adolescent PMTCT and HIV programming as adolescents were contributing disproportionally to HIV transmission. One of the local implementing partners Mothers2Mothers was running a mentoring program so the team wanted to examine the barriers and challenges faced by adolescents to participating in the program. The research was conducted by a local university research team in partnership with the ministry and implementing partner. Study objectives: 1. Examine the beliefs, perceptions, social norms, and behaviours among pregnant and postnatal HIV+ adolescents in programme-supported districts surrounding the national PMTCT programme and mentor mother services 2. Describe perceived needs of pregnant and postnatal HIV+ adolescents in program-supported districts with regard to the national PMTCT programme and mentor mother services 3. Identify perceived barriers for pregnant and postnatal HIV+ adolescents to uptake/participation and retention in the national PMTCT programme and mentor mother services in program-supported districts in Malawi *Methods:* Qualitative focus group discussions with HIV-positive pregnant and postpartum adolescents who participated and did not participate in the mentor mothers program in program-supported districts. *Results:* Examined needs of HIV-positive adolescents within a mentoring support program. Based on study results, the mentor program hired and trained Adolescent Champions (same age peers) within 3 months of study completion to provide special services for adolescents in the program [31].	

In addition to monitoring program results, we have also documented government implementers’ and local research partners’ [26] experiences in the program. Results suggest positive responses to participation in the process. Table 3 cites two quotes from a project in Pakistan, one from before the IR and one after, which exemplify the common responses seen from participants, and in particular highlight the co-production of knowledge.

**Table 3:** Quotes from participants in embedded implementation research in Pakistan [27]

**Before:** “Researchers don’t help us at all. The research they produce is irrelevant to us. It never helps solve our problems. They never talk to us. And we never see the results of research. All they want to do is publish their paper.” *Pakistan District EPI manager* **After:** “Every year, three to five thick reports with a long list of recommendations are brought on my table, I seldom get a chance to skim through them, but this time, I am co-producing the required knowledge….” *Pakistan District EPI manager*	

### Funding embedded implementation research at UNICEF

To date, UNICEF-supported IR studies have been funded almost exclusively through projects, as part of the program monitoring or learning agenda. Studies are typically short-term and require limited funding. For example, five HIV-related IR studies in 2017 cost US$15–35,000 each for data collection and analysis and were completed within 5 months. Projects to date range from $10,000 to $70,000 (usually $20,000-$40,000) and 5 to 18 months (usually 12 months). This near real-time aspect of embedded IR is recognized as one of the advantages as research results can be available within planning cycles for decision making and program adaptation. While the research projects themselves generally require limited funding, building local capacity to run the studies, both for the implementers and local researchers, often requires additional resources beyond the actual research costs for training and technical assistance during the studies, consistent with building capacity of research co-production as discussed by Agyebong and colleagues [8].

### Key challenges and considerations in developing UNICEF’s embedded IR approach

IR has been recognized as critical to strengthen health systems [14, 27, 28]. However, the concept of embedding research into real-world policy, practice, and implementation is somewhat new in the field of global children’s programming, and uptake of the approach has challenges. For example, we have found that in-country partners, including local and national-level government counterparts, and some donors, need to be convinced about the value of IR. By engaging stakeholders in this approach, we have seen a recognition of the use of IR for program planning and enthusiasm for continuation of the implementer-researcher partnerships and research co-production after completion of the initial IR project. Also, for IR to be truly country driven, donors and partners have to trust that countries can identify the most relevant implementation barriers, transforming them into questions to investigate. This will require adaptations for review of research proposals, which may be more undefined regarding objectives and methods, given that these will be defined as a first step of the research process. Greater emphasis on domestic resource mobilization for embedding IR into the decision-making process and into routine program funding is needed. This problem can be overcome by advocacy, showcasing the value IR brings, building the capacity of partners on IR, and engaging them early and throughout the research activities so as to address their stated priorities, gaps in knowledge, and improve policy and ownership. Weighing opportunity costs between investing in service delivery and/or implementation research requires continued focus, as turnover of leadership and staff can reverse gains made in many contexts.

Another challenge is that in some cases, our partner implementers and researchers have been overly ambitious or wanted to pursue larger scale research. However, our experiences show that time-limited, small-scale and relatively inexpensive IR studies can lead to important learnings that have translated into changes in policies or approaches. We saw that the IR brought these two communities closer together with the benefits of greater relevance of research to programming, introduction of new methods, and faster implementations of specific solutions. To build incentives for researchers to do IR, showing how their research led to policy and practice change and not just traditional peer reviewed publications could be valued by universities as a component for promotion or research funding. Recent programs that promote and fund partnerships for implementation and health systems research, such as those by the Australian and UK Medical Research Councils and the Doris Duke Foundation African Health Initiative, have been a welcome contribution to the funding landscape [29, 30].

Assessment of research quality and how the results are contributing to the existing evidence base also needs to be addressed. Many IR studies while being used locally for in country program improvements may not be published in peer-review literature, but nevertheless could contribute to expanding the evidence base on implementation strategies. Therefore, publishing in on-line platforms, such as the TDR Gateway (https://www.who.int/tdr/publications/tdr-gateway/en/) or similar sites, will allow for quality-assurance and rapid wider dissemination.

## Conclusion

UNICEF has built on the work of The Alliance, TDR, and others, to adopt an innovative embedded IR approach to meet country program needs to assure the right to health and well-being “For Every Child.” Implementation research at UNICEF is now supported across several sectors and by the Office of Research, suggesting a sustainable future for the approach. In addition, program staff from more than 25 countries have received training on this approach and how to support it with their partners during program implementation. IR has also been added to the UNICEF-University of Melbourne-Nossal Institute Health Systems Research Massive Open Online Course (https://www.futurelearn.com/courses/health-systems-strengthening). We have also seen an expansion of global partners and universities supporting this research, such as the Implementation Research and Delivery Science Coalition (https://www.harpnet.org/wp-content/uploads/2018/10/Coalition-Statement.pdf) and several TDR Postgraduate programs (https://www.who.int/tdr/capacity/strengthening/postgraduate/en/) in Bangladesh, Zambia, and Ethiopia, have developed partnerships with local UNICEF country programs.

Embedding research into local systems and service delivery can address concerns of implementers and support selection of effective implementation strategies, taking into account local context and systems complexity to address implementation barriers. UNICEF embedded IR seeks to understand how to overcome these barriers within maternal, newborn, child, and adolescent programs—in and beyond the health sector. In addition, it can be used to test applicability of approaches in different contexts (e.g., geographic, political, physical environment, social, economic). Ultimately, the aim of these activities is to build embedded IR capacity and accelerate large-scale adoption, effective implementation, and dissemination of successful approaches that generate results for women and children.

## Data Availability

Not applicable.
